# LLM-based assessment of HTTPS cybersecurity awareness: Dataset from moroccan web users and webmasters

**DOI:** 10.1016/j.dib.2025.112024

**Published:** 2025-09-04

**Authors:** Abdelhadi Zineddine, Abdeslam Rehaimi, Mohamed Zaoui, Yousra Belfaik, Yassine Sadqi, Said Safi

**Affiliations:** a Laboratory LIMATI, Polydisciplinary Faculty, Sultan Moulay Slimane University (USMS), Beni-Mellal 23000, Morocco; b Laboratory ISIMA, Polydisciplinary Faculty, Ibn Zohr University (UIZ), Taroudant 83000, Morocco; c Laboratory L2IS, Faculty of Sciences and Techniques, Cadi Ayyad University (UCA), Marrakesh 40000, Morocco

**Keywords:** Web security practices, Web-user behavior, Cybersecurity risk behaviors, Awareness level, LLM-Based interpretation, HTTPS deployment

## Abstract

Cybersecurity awareness plays a fundamental role in protecting digital communications, particularly in the deployment and use of the HTTPS protocol. While previous studies have explored website security practices, there is a lack of available datasets that empirically assess both awareness levels and implementation behaviors of web-users and website administrators. This dataset addresses this gap by analyzing cybersecurity awareness and HTTPS-related behaviors of 440 Moroccan voluntary participants, including web users and webmasters. Data was collected via a structured Google Forms survey, disseminated through web development and cybersecurity communities on online platforms such as Facebook, WhatsApp and LinkedIn.

The responses collected from multiple-choice questions (MCQs) and free-text entries (categorized using the GPT-4o large language model (LLM)) were pre-processed and score-encoded according to a predefined mapping scheme. Participants’ awareness levels were classified as Low, Moderate, or High on total scores. To identify behavioral patterns, the unsupervised KMeans clustering algorithm was applied separately to user and webmaster groups. Principal Component Analysis (PCA) and LLM-based interpretation provided insights into awareness profiles and cybersecurity risk behaviors.

The dataset includes raw survey responses, score-encoded data, clustering outputs, and LLM-generated awareness assessment reports. It serves both as supplementary material for a novel hybrid cybersecurity assessment methodology for HTTPS deployment presented in [1], and as a standalone resource for researchers and practitioners examining HTTPS usage, certificate management, and behavioral risk profiling. This dataset is a valuable asset for empirical research and practical improvements in cybersecurity awareness within role-based and regional web ecosystems.

Specifications Table

**Table:** 

Subject	Computer Sciences
Specific subject area	Cybersecurity awareness and HTTPS-related behaviors of web users and webmasters, with focus on secure browsing and HTTPS deployment practices.
Type of data	Table, Spreadsheet, Text file, JSON
Data format	Raw, Processed, Encoded, Clustered, Analyzed, Interpreted
Data collection	Data were collected from 440 voluntary Moroccan participants through a structured Google Forms survey consisting of 11 questions, including multiple-choice and free-text responses [2]. Python scripts were used for data preprocessing and score encoding. Awareness levels were categorized based on total calculated scores. KMeans clustering and Principal Component Analysis (PCA) were applied to identify behavioral patterns, followed by interpretive analysis using the GPT-4o large language model.
Data source location	Morocco
Data accessibility	Repository name: Mendeley Data Data identification number (DOI): 10.17632/g3fbm5pwm6.3 Direct URL to data: https://data.mendeley.com/datasets/g3fbm5pwm6/3
Related research article	A. Zineddine, Y. Belfaik, Y. Sadqi, S. Safi, A Novel Hybrid Cybersecurity Assessment Methodology for HTTPS Deployment, High Confidence Computing (2025). https://doi.org/10.1016/j.hcc.2025.100344.

## 1 Value of the Data


 

• This dataset provides unique empirical evidence that jointly assesses cybersecurity awareness and practical HTTPS deployment behaviors among two key roles: general web users and webmasters [3]. As the first publicly available dataset of its kind focused on the Moroccan web ecosystem, it fills a critical gap in the cybersecurity awareness literature, particularly in regions where such behavioral data is scarce or non-existent [4] .
• The dataset is designed with common questions for both web users and webmasters, enabling direct comparisons of awareness and behavior across roles. Additionally, it includes technical items specific to webmasters (e.g., SSL certificate configuration methods and expiry management practices), offering insights into potential HTTPS misconfiguration issues [5,6]. This structure allows researchers, educators, and policymakers to explore secure browsing habits, identify awareness gaps, and evaluate real-world HTTPS deployment strategies from both user and developer perspectives [7].
• The dataset includes pre-processed, score-encoded responses and KMeans clustering results with PCA projections. This allows researchers to apply and validate various unsupervised learning and pattern recognition techniques on real-world security-related survey data [8].
• The dataset incorporates interpretive outputs generated using GPT-4o, making it valuable for studies exploring the integration of large language models in behavioral data interpretation and cybersecurity awareness assessment [9,10].
• Given the specific regional scope (i.e., Moroccan web ecosystem) and the behavioral variables captured, the data can support localized policy-making, awareness campaigns, and cross-cultural comparative research.
• Researchers and practitioners can reuse the dataset to develop training material, awareness assessment tools, or simulations for evaluating secure browsing behavior and HTTPS deployment strategies [11].

## 2 Background

The primary motivation behind compiling this dataset was to support the development and validation of our novel hybrid cybersecurity assessment methodology for HTTPS deployment [1]. This methodology combines three core cybersecurity assessment techniques: examination, testing, and interviewing [12]. While examination and testing require structured technical data (e.g., related to web server configurations [13] or X.509 certificate attributes [14]), the interviewing component necessitates reliable, structured information on the awareness and behaviors of both web users and webmasters regarding HTTPS practices.

To address this requirement, a structured survey [2] was designed and distributed among Moroccan web users and webmasters to collect empirical data concerning their knowledge, behaviors, and practices related to secure web browsing and HTTPS deployment. The resulting dataset captures a wide range of awareness indicators and implementation insights, enabling the behavioral analysis necessary for the interviewing component of the methodology [15,16].

This Data in Brief article complements the related research article submitted to High Confidence Computing [1] by providing an in-depth description of the dataset used. It documents the survey structure, encoding logic, processing pipeline, and analytical outputs (e.g., clustering and LLM-based interpretation) in detail, facilitating reproducibility and encouraging reuse by the broader cybersecurity research community.

## 3 Data Description

The dataset is based on a structured online survey designed to assess cybersecurity awareness and HTTPS-related behaviors among web users and webmasters in Morocco [2]. The survey comprises 11 questions (Q1–Q11), of which the first five questions (Q1–Q5) are shared between both groups and aim to evaluate general awareness and secure browsing practices, such as concern for website security (Q1), understanding the difference between HTTP and HTTPS (Q2), preference for HTTPS or HTTP (Q3), attention to browser security alerts (Q4), and engagement with HTTPS lock indicators (Q5). The remaining six questions (Q6–Q11) target webmasters specifically and cover HTTPS configuration practices (Q6–Q7), knowledge of SSL certificate types (Q8), and responses regarding the types of certificates used (Q9), certificate expiration awareness (Q10), and certificate validation practices (Q11). Questions Q9 and Q11 are open-ended and were processed and categorized using the GPT-4o large language model, as described in the following section. A full list of the questions and their formats is included in the file survey_questions.pdf provided in the dataset repository [3]. The survey was administered via Google Forms and disseminated through Moroccan online communities focused on cybersecurity and web development, ensuring voluntary and diverse participation.

Several datasets are provided as supplemental files in the Mendeley Data repository [3]. The core data are available in comma-separated values (.csv) format, including Collected_dataset.csv, which contains the original raw responses from 440 Moroccan participants (comprising 359 web users and 81 webmasters), and Encoded_collected_dataset.csv, which contains score-based encodings derived from a predefined scoring schema documented in Score_Map_Used.xlsx and Dataset_dictionary.xlsx. This mapping file outlines how each survey response was transformed into a numerical score for analysis.

Processed datasets are further divided by role: Users_awareness_clusters.csv and Webmasters_awareness_clusters.csv. These files include additional computed attributes, such as the overall awareness level, which is classified as Low, Moderate, or High based on the total score, and cluster labels assigned through an unsupervised KMeans clustering algorithm.

Clustering results and dimensionality reduction coordinates are available in JSON format (User_cluster_mean_values.json*,* User_pca_coordinates.json, etc.), ensuring transparency into model outputs. Additionally, interpretive assessment reports generated using the GPT-4o large language model are included as human-readable text files (LLM_assessment_report_users.txt, LLM_assessment_report_webmasters.txt). All files are organized into well-defined subfolders, as illustrated in Table 1 bellow, and are compatible with standard analysis tools such as Microsoft Excel, Python, and R. This modular structure facilitates reproducibility and ease of use for both qualitative and quantitative analysis.

**Table 1:** Overview of the dataset components with descriptions of their content and roles.

Folder Name	File Name with Extension	Description	Role
Documentation_ and_Metadata	Dataset_ dictionary.xlsx	Describes all survey variables, question labels, encoding values, and awareness level scoring criteria.	Helps users interpret dataset structure and scoring methodology.
	README.txt	Primary documentation file outlining the dataset’s purpose, content structure, and usage guidelines.	Entry point to understanding the dataset and its organization.
Collected_data	Collected_ dataset.csv	Raw survey responses from 440 Moroccan participants (web users and webmasters), including 11 questions (5 common, 6 specific to webmasters). Responses consist of both MCQ and free-text entries.	Provides unprocessed data for baseline analysis or replication.
	survey_ questions.pdf	Full PDF version of the administered questionnaire detailing question phrasing and format.	Enables replication or extension of the survey instrument.
	survey_ link.txt	Contains the public URL of the original Google Form used to collect data.	For consultation, future participation or awareness-raising.
Preprocessed_ data	Encoded_ collected_ dataset.csv	Dataset with encoded and scored responses converted from raw data based on a predefined scheme.	Enables numerical analysis and clustering.
	Score_map_ used.xlsx	Defines encoding and scoring rules used to convert text responses into numerical values.	Supports reproducibility of awareness level scoring and preprocessing pipeline.
Final_datasets	Users_awareness_ clusters.csv	Encoded dataset for general web users, including awareness level classification and clustering output.	Final processed data for analysis of user group behavior.
	Webmasters_ awareness_ clusters.csv	Encoded dataset for webmasters, including awareness level classification and clustering output.	Final processed data for analysis of webmaster group behavior.
Clustering_ outputs	User_cluster_ mean_values.json	Cluster-wise average scores for users, computed after KMeans clustering.	Input for LLM-based interpretation.
	Webmaster_cluster_ mean_values.json	Cluster-wise average scores for webmasters.	Input for LLM-based interpretation.
	User_pca_ coordinates.json	PCA projections of user clusters for dimensionality reduction.	Input for LLM-based interpretation, highlighting internal behavioral patterns within clusters.
	Webmaster_pca_ coordinates.json	PCA projections of webmasters	
LLM_outputs	LLM_assessment_ report_users.txt	GPT-4o-generated interpretive summary of cybersecurity behaviors and awareness trends for web users clusters.	Provides awareness assessment summaries that support human interpretation and deeper analysis of clustering outcomes using GPT-4o as LLM.
	LLM_assessment_ report_ webmasters.txt	GPT-4o-generated interpretive summary of cybersecurity behaviors and awareness trends for webmaster clusters.	

## 4 Experimental Design, Materials and Methods

**Fig. 1:**
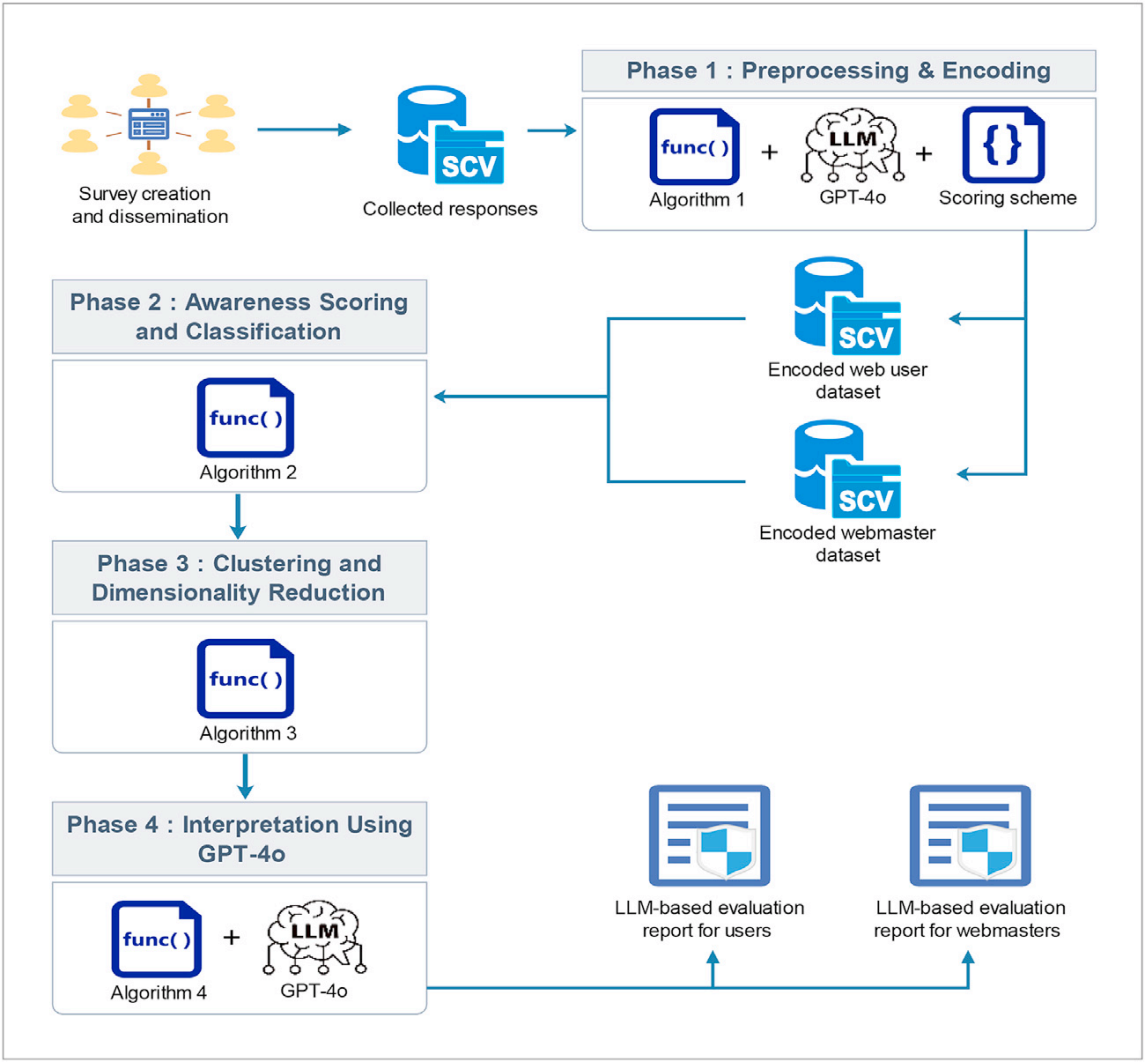
Workflow of the Experimental Design and Analytical Processing for the HTTPS Awareness Dataset.

Following the design and distribution of a structured online survey via the Google Forms platform, responses were collected from Moroccan web users and webmasters through targeted dissemination in online communities focused on web development and cybersecurity. After exporting the collected responses in CSV format, the workflow was structured as illustrated in the Fig. 1 into four main phases, each contributing to the experimental design and analytical processing for the HTTPS awareness dataset.

### 4.1 Phase 1: preprocessing and encoding

This initial phase focused on preparing the raw survey data for further analysis. The process began with data cleaning and formatting to ensure consistency and compatibility, including the standardization of free-text inputs and the removal of incomplete or invalid entries.

**Fig. 2:**
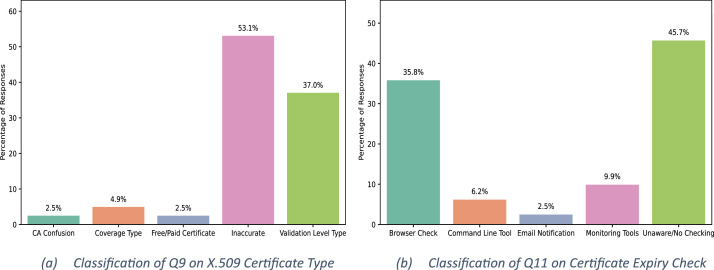
Semantic Categorization of Open-Ended Survey Responses Using GPT-4o.

Two open-ended questions (Q9 and Q11) were semantically classified using the GPT-4o language model, guided by a structured prompt. A Python script (described in Algorithm 1) automated the classification of these responses into predefined semantic categories. The classification outputs are visualized in Fig. 2, with subfigure (a) representing Q9 and subfigure (b) representing Q11.

**Algorithm 1:** GPT-4o-Based Classification and Encoding of Webmaster’s Free-Text Responses.

**Input**: CSV file with survey responses for Q9 and Q11, OpenAI API Key
**Output**: Normalized and encoded categories for Q9 and Q11 appended to the dataset
1: //**Step 1: Dataset Initialization**
2: Load Collected_dataset.csv into DataFrame df
3: Identify column Q9 (certificate type) and Q11 (expiry check method)
4: //**Step 2: Define GPT Classifier for Q9 (Certificate Type)**
5: **for all** response in column Q9**do**
6: Construct prompt with 5 categories: Validation Level, Free/Paid, Coverage Type, CA Confusion, Inaccurate
7: Call GPT-4o API with classification prompt
8: Store result in column Q9_GPT_Category
9: **end for**
10: //**Step 3: Normalize and Encode Q9 Category**
11: **for all** entry in Q9_GPT_Category **do**
12: **if** entry contains category keyword**then**
13: Normalize to matching label
14: **else**
15: Assign "Inaccurate"
16: **end if**
17: Encode using: Validation Level= 4, Free/Paid= 3, Coverage Type= 2, CA Confusion = 1, Inaccurate = 0
18: Save in Q9_GPT_Certificate_Type_Encoded
19: **end for**
20: //**Step 4: Define GPT Classifier for Q11 (Expiry Check Method)**
21: **for all** response in column Q11**do**
22: Construct prompt with 5 categories: Browser Check, Command Line Tool, Monitoring Tools, Email Notification, Unaware
23: Call GPT-4o API with classification prompt
24: Store result in column Q11_GPT_Category
25: **end for**
26: //**Step 5: Normalize and Encode Q11 Category**
27: **for all** entry in Q11_GPT_Category **do**
28: **if** entry contains keyword (e.g., browser, email, monitor)**then**
29: Normalize to matching label
30: **else**
31: Assign "Unaware/No Checking"
32: **end if**
33: Encode using: Browser Check= 4, Command Line= 3, Monitoring= 2, Email Notification= 1, Unaware= 0
34: Save in Q11_GPT_Certificate_Expiry_Check_Encoded
35: **end for**
36: //**Step 6: Export Updated Dataset**
37: Save df as Encoded_collected_dataset.csv

All responses, including both multiple-choice and categorized free-text answers, were numerically encoded using a predefined scoring scheme. The resulting dataset was then split into two role-specific CSV files: one for general web users and another for webmasters.

### 4.2 Phase 2: awareness scoring and classification

For each participant, a total awareness score was calculated by summing the encoded values across all relevant survey questions. Based on these cumulative scores, participants were categorized into one of three predefined awareness levels: Low, Moderate, or High. This classification process was automated using a structured Python script, as detailed in Algorithm 2.

**Algorithm 2:** Awareness Scoring and Classification of Survey Participants.

**Input**: Encoded web user and webmaster datasets (based on Score_Map_Used.xlsx)
**Output**: Awareness level category and encoded value stored in each dataset
1: **// Step 1: Load Pre-Encoded Datasets**
2: Load encoded dataset for web users
3: Load encoded dataset for webmasters
4: **// Step 2: Compute Awareness Scores**
5: **for all**user participants**do**
6: Sum encoded values from Q1 to Q5 into column Awareness_Score
7: **end for**
8: **for all**webmaster participants**do**
9: Sum encoded values from Q1 to Q11 into column Awareness_Score
10: **end for**
11: **//Step 3: Define Role-Specific Classification Function**
12: **function** classify(score, role)
13: ** if**role = user**then**
14: ** if**score ≤ 3**then return**"Low"
15: ** else if**score ≤ 6**then return**"Moderate"
16: ** elsereturn**"High"
17: ** end if**
18: ** else if**role = webmaster**then**
19: ** if**score ≤ 6**then return**"Low"
20: ** else if**score ≤ 12**then return**"Moderate"
21: ** elsereturn**"High"
22: ** end if**
23: ** end if**
24: **end function**
25: **//Step 4: Apply Classification and Numerical Encoding**
26: **for all**participants in user dataset**do**
27: Apply classify(score, "user") to Awareness_Score
28: Map classification to corresponding code: ”Low”→0, ”Moderate”→1, ”High”→2
29: Store the numeric code in Awareness_Level_Encoded
30: **end for**
31: ** for all**participants in user dataset**do**
32: Apply classify(score, "webmaster") to Awareness_Score
33: Map classification to corresponding code: ”Low”→0, ”Moderate”→1, ”High”→2
34: Store the numeric code in Awareness_Level_Encoded
35: **end for**

### 4.3 Phase 3: clustering and dimensionality reduction

**Fig. 3:**
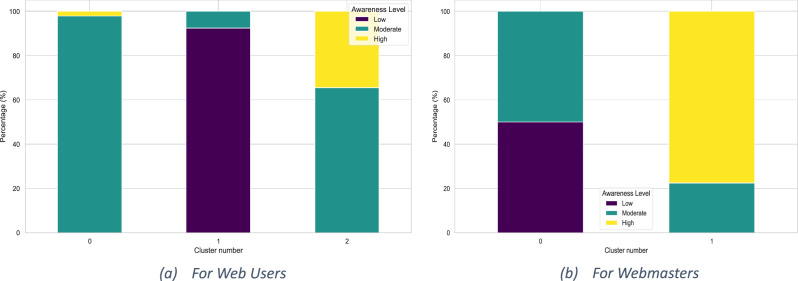
Awareness-Level Distribution Across KMeans-Identified Clusters.

To uncover patterns within the participant responses, unsupervised KMeans clustering was performed separately for web users and webmasters. The detailed procedure is outlined in Algorithm 3, which includes feature scaling, identification of the optimal number of clusters using silhouette analysis, and the assignment of cluster labels to participants. For each cluster, mean values were computed to characterize representative behavioral profiles. Additionally, Principal Component Analysis (PCA) was applied to reduce the dimensionality of the data, enabling effective visualization of clustering results. The distribution of awareness levels across clusters is illustrated in Fig. 3, with subfigures (a) for web users and (b) for webmasters.

**Algorithm 3:** Clustering and Dimensionality Reduction of Web User and Webmaster Responses.

**Input**: Preprocessed and scored datasets for web users and webmasters
**Output**: Cluster labels, PCA projection, JSON outputs for interpretive analysis
1: **//Step 1: Feature Selection and Scaling**
2: **for all**roles in {user, webmaster} do
3: **if**role = user **then**
4: Load Users_awareness_clusters.csv
5: Define feature set: Q1 to Q5 + Awareness_Score
6: **else**
7: Load Webmasters_awareness_clusters.csv
8: Define feature set: Q1 to Q11 + Awareness_Score
9: **end if**
10: Extract features matrix X from dataset
11: ApplyStandardScaler to normalize values → X_scaled
12: **//Step 2: Determine Optimal Number of Clusters**
13: Initialize: best_k_silhouette ← 2, best_score ← −1
14: **for***k*in candidate range**do**
15: Fit KMeans with*k*clusters on X_scaled
16: Compute silhouette_score
17: **if**current score > best score**then**
18: Update best_k_silhouette and best_score
19: **end if**
20: **end for**
*21:* Visualize silhouette scores to confirm optimal*k*
22: **//Step 3: Apply KMeans Clustering**
23: Fit KMeans with best_k_silhouette
24: Assign cluster labels to <Role>_cluster column
25: Compute cluster-wise mean values to characterize each group
26: **//Step 4: Dimensionality Reduction using PCA**
27: Apply PCA with 2 components to X_scaled → X_pca
28: Create DataFrame pca_df with PC1, PC2, cluster ID, and awareness level
29: **//Step 5: Visualization**
30: Calculate percentage distribution of awareness levels
31: Plot the 2D PCA projection (clusters colored and awareness level styled)
32: Annotate awareness distribution on the plot
33: **//Step 6: Save Outputs & Export JSON Files**
34: Save updated dataset with cluster labels to <Role>_awareness_clusters.csv
35: Export cluster-wise mean values to <Role>_ cluster_mean_values.json
36: Export PCA coordinates to <Role>_ pca_coordinates.json
37: **end for**

### 4.4 Phase 4: interpretation using GPT-4o

Cluster metrics and PCA coordinates were exported in JSON format to serve as structured inputs for interpretive analysis using the GPT-4o large language model. Leveraging prompt engineering techniques, the model was guided to generate human-readable assessment reports that qualitatively describe behavioral patterns and cybersecurity awareness characteristics across identified clusters. The automated interpretation process is detailed in Algorithm 4.

**Algorithm 4:** Interpretive Analysis of Clustering Results Using GPT-4o.

**Input**: Cluster-wise mean values and PCA coordinates in JSON format
**Output**: Human-readable interpretation reports for each role, generated by GPT-4o
1: **for all** roles in {user, webmaster}**do**
2: **// Step 1: Load Structured Data**
3: Load <Role>_ cluster_mean_values.json
4: Load <Role>_ pca_coordinates.json
5: //**Step 2: Construct the Structured Prompt**
6: Define role-specific task instructions for GPT-4o including:
7: • Interpretation of cluster-wise mean values and awareness scores
8: • Interpretation of PCA projections and visual distribution
9: • Differentiation of participant behaviors and cybersecurity practices
10: • Identification of insecure behaviors or patterns
11: • Comparative analysis between clusters
12: • Summary of findings and suggested recommendations
13: Embed the first few JSON records into the prompt
14: **//Step 3: Send Prompt to GPT-4o**
15: Submit prompt to GPT-4o via OpenAI API
16: Retrieve the generated textual interpretation
17: **//Step 4: Save Interpretation Output**
18: Save the GPT-4o interpretation to LLM_assessment_report_<Role>.txt
19: **end for**

All data processing, clustering, and interpretive procedures were executed using Python 3.12.7, employing libraries such as pandas for data handling, scikit-learn for clustering and dimensionality reduction, matplotlib for visualization, and the OpenAI API for interaction with the GPT-4o model.

## Limitations

While this dataset provides valuable empirical insights into cybersecurity awareness and HTTPS deployment practices among Moroccan web users and webmasters, a few contextual considerations should be acknowledged. The survey was disseminated through online platforms, which may have attracted participants already involved in digital or cybersecurity communities, potentially resulting in a sample with higher-than-average awareness levels compared to the broader population.

Although the dataset includes a variety of respondent profiles, it is geographically limited to Morocco and may reflect region-specific cultural or infrastructural characteristics. However, the methodology is reproducible and can be applied in other regions to enable comparative studies and broader generalization.

Open-ended responses were categorized using GPT-4o, a large language model that enables scalable and consistent interpretation. While effective, this approach may introduce subtle variations in classification depending on the phrasing of prompts or the use of alternative LLMs.

These considerations do not diminish the dataset’s analytical value but rather highlight opportunities for future research, replication, benchmarking, and contextual adaptation.

## Ethics Statement

The dataset was collected via an online survey with entirely voluntary participation. To ensure participant privacy, no personal or sensitive information (e.g., names, email addresses, IP addresses, or geolocation data) was collected at any stage of the survey process.

Participants were clearly informed that (1) no identifying data would be collected and that their participation would remain fully anonymous, and (2) their responses would be used exclusively for scientific research purposes in the field of cybersecurity. The survey’s design and dissemination complied with ethical standards and adhered to the data redistribution policies of the platforms through which it was shared (e.g., Facebook, WhatsApp, LinkedIn).

## Credit Author Statement

**Abdelhadi Zineddine**: Conceptualization, Methodology, Software, Investigation, Data Curation, Writing - Original Draft; **Abdeslam Rehaimi**: Methodology, Software, Investigation, Data Curation, Writing - Original Draft; **Mohamed Zaoui**: Methodology, Formal analysis, Investigation, Data Curation, Visualization, Writing - Original Draft; **Yousra Belfaik**: Methodology, Formal analysis, Investigation, Visualization; **Yassine Sadqi**: Conceptualization, Methodology, Validation, Visualization, Supervision; **Said Safi**: Formal analysis, Validation, Visualization, Supervision.

## Data Availability

Mendeley DataCybersecurity Awareness Dataset on HTTPS Usage among Moroccan Web Users and Webmasters (Reference data)

## References

[1] A. Zineddine, Y. Belfaik, Y. Sadqi, S. Safi, A novel hybrid cybersecurity assessment methodology for HTTPS deployment, high confidence computing (2025). 10.1016/j.hcc.2025.100344.

[2] Survey, Evaluation of Moroccan HTTPS websites. https://forms.gle/kiZWQuUXWNpHLVCu7, 2025 (accessed 18 July 2025).

[3] A. Zineddine, A. Rehaimi, Y. Belfaik, Y. Sadqi, S. Safi, Cybersecurity awareness dataset on HTTPS usage among Moroccan web users and webmasters, Mendeley Data, V3, 2025. 10.17632/g3fbm5pwm6.3. PMC12545824 41143264

[4] Fartitchou M., Lamaakal I., Makkaoui K.E., Allali Z.E., Maleh Y. (2025). BlockMEDC: blockchain smart contracts system for securing Moroccan higher education digital certificates. IEEE Access.

[5] Fahl S., Acar Y., Perl H., Smith M. (2014). Proceedings of the 9th ACM Symposium on Information, Computer and Communications Security.

[6] Sadqi Y., Maleh Y. (2022). A systematic review and taxonomy of web applications threats. Inform. Security J..

[7] Zaoui M., Yousra B., Yassine S., Yassine M., Karim O. (2024). A comprehensive taxonomy of social engineering attacks and defense mechanisms: toward effective mitigation strategies. IEEE Access.

[8] Lamaakal I., Essahraui S., Maleh Y., Makkaoui K.E., Ouahbi I., Bouami M.F., El-Latif A.A.A., Almousa M., Peng J., Niyato D. (2025). A comprehensive survey on tiny machine learning for Human behavior analysis. IEEE Internet Things J..

[9] Malhotra A., Jindal R. (2024). XAI transformer based approach for interpreting depressed and suicidal user behavior on online social networks. Cogn. Syst. Res..

[10] Khader M., Karam M., Fares H. (2021). Cybersecurity Awareness Framework for Academia. Information.

[11] Rehaimi A., Sadqi Y., Maleh Y., Gaba G.S., Gurtov A. (2024). Towards a federated and hybrid cloud computing environment for sustainable and effective provisioning of cyber security virtual laboratories. Expert Syst. Appl..

[12] Scarfone K., Souppaya M., Cody A., Orebaugh A. (2008). Technical guide to information security testing and assessment. NIST Special Public..

[13] Zineddine A., Chakir O., Sadqi Y., Maleh Y., Singh Gaba G., Gurtov A., Dev K. (2024). A systematic review of cybersecurity assessment methods for HTTPS. Comput. Electr. Eng..

[14] Zulfiqar M., Janjua M.U., Hassan M., Ahmad T., Saleem T., Stokes J.W. (2022). Tracking adoption of revocation and cryptographic features in X.509 certificates. Int. J. Inf. Secur..

[15] Mohammed M.A., Hamid R.A., AbdulHussein R.R. (2024). Data collection and preprocessing in web usage mining: implementation and analysis. Iraqi J. Comput. Informat..

[16] Zineddine A., Sadqi Y. (2024). Digital Technologies and Applications.

